# Case Report: Torsade de Pointes Induced by the Third-Generation Epidermal Growth Factor Receptor–Tyrosine Kinase Inhibitor Osimertinib Combined With Litsea Cubeba

**DOI:** 10.3389/fcvm.2022.903354

**Published:** 2022-05-31

**Authors:** Xia-yan Zhang, Cha-bin Wu, Cai-xia Wu, Li Lin, Yue-juan Zhou, Yan-yan Zhu, Wei-qiang Tian, Song-mei Luo

**Affiliations:** ^1^Department of Pharmacy, Lishui Central Hospital, Lishui, China; ^2^Department of Pharmacy, Longquan Hospital of TCM, Lishui, China; ^3^Department of Pharmacy, Traditional Chinese Medicine Hospital of Qingyuan, Lishui, China; ^4^Department of Cardiovascular Medicine, Lishui Central Hospital, Lishui, China

**Keywords:** torsade de pointes, osimertinib, Litsea Cubeba, QT prolongation, adverse events

## Abstract

Torsades de Pointes (TdP) occurred in a 68-year-old female with epidermal growth factor receptor (EGFR) mutant lung cancer administered osimertinib, the third-generation EGFR tyrosine kinase inhibitor (TKI). Electrocardiogram (ECG) recorded at Tdp showed QT prolongation (QTc = 515 ms), to which a Traditional Chinese Medicine (TCM) named “Litsea Cubeba” may have contributed. After discontinuation of osimertinib and Litsea Cubeba, magnesium supplementation, potassium supplementation, lidocaine infusion, and the pacemaker frequency adjustment, Tdp terminated. However, QT prolongation sustained at discharge (QTc = 528 ms), partly because of the emergency use of amiodarone. Osimertinib may prolong the QT interval leading to TdP, especially when multiple risk factors to lengthen QT interval are incidentally overlapped. Thus, regular monitoring of ECG and appropriate management of concomitant drugs are highly recommended.

## Introduction

Osimertinib is an oral, irreversible, third-generation epidermal growth factor receptor (EGFR) tyrosine kinase inhibitor (TKI), recommended as a first-line treatment for EGFR-mutated advanced or metastasis non-small cell lung cancer (NSCLC) (1, 2). Since vascular and cardiac potassium channels are regulated largely by EGFR tyrosine kinase (3), cardiotoxicity such as congestive heart failure and QT prolongation has been reported to be common with Osimertinib. Of the 1,479 patients treated with TAGRISSO in clinical trials (4, 5), 0.8% were found to have a QTc >500 ms, and 3.1% of patients had an increase from baseline QTc >60 ms, but no QTc-related arrhythmias were reported. Thus, osimertinib-induced QT prolongation leading to the possible development of Torsades de Pointes (TdP) has received less attention. Here we present a 68-year-old woman with advanced NSCLC (T1N2M0, IIIa) had sudden TdP, which was caused by osimertinib combined with Traditional Chinese Medicine (TCM) named “Litsea Cubeba.”

## Case Description

A 68-year-old woman, who had a history of lung adenocarcinoma for 1 year, was admitted to our hospital with “syncope” on January 02, 2022. In April 2020, she visited our hospital due to waist pain and back pain. The Chest CT on 15 April 2020 showed space-occupying lesions in the left upper lung, which was considered to be the lung cancer, accompanied by pleural effusion in both sides. The PET-CT on 20 April 2020 showed subpleural solid nodules in the left upper lung with FDG metabolism slightly elevated, which was considered to be Pulmonary CA. The target scan of pulmonary nodules on 23 April 2020 showed Subpleural solid nodule in the left upper lung and ground-glass nodule in the left lower dorsal segment. Then she underwent left total pneumonectomy in the first affiliated hospital of Zhejiang University, with post-operative pathology showing the stage was T1N2M0, stage IIIA. Genetic testing showed that exon 21 EGFR-L858R was positive with a mutation frequency of 1.65%. She was given lcotinib (Conmana) 125 mg tid for targeted therapy, and had a dose reduction or discontinuation due to diarrhea and itch according to the instructions of dosage adjustment. On 13 October 2021, re-examination of PET-CT showed vertebral metastasis in the S1 vertebral body. On 20 October 2021, a second genetic testing: exon 20 EGFR -T790M was positive with a mutation frequency of 0.75%. Then, the treatment was adjusted to osimertinib (TAGRISSO) 80 mg qd in 1 November 2021.

**FIGURE 1:**
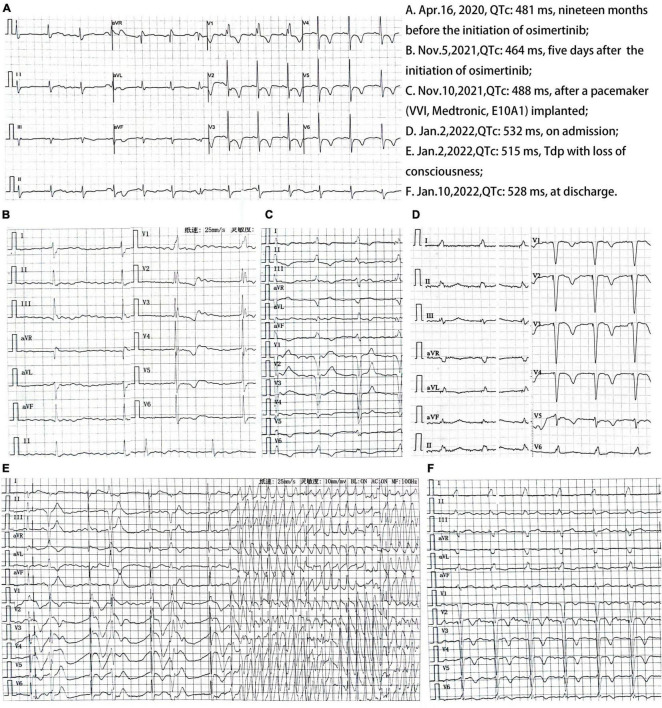
Twelve-lead electrocardiogram (ECG).

Her transthoracic ultrasound cardiography (UCG) was not examined at the initiation of osimertinib, but it showed a left ventricular end-diastolic diameter (LVEDD) of 54 mm and a left ventricular ejection fraction (LVEF) of 60% in 10 months before the initiation on 1 January 2021 and showed a LVEDD of 57 mm and an LVEF of 62% 10 days after the initiation on 11 November 2021. She had >1 year history of atrial fibrillation with third degree atrioventricular blocker, but no provable electrocardiogram (ECG) evidence could be provided. And we found no indications in her ECG [QTc (QT interval corrected by Bazetts’s formula, QTc = QT/RR^0.5^): 481 ms, HR: 76 bpm, Figure 1A] examined 19 months before. Because of some personal reasons, her ECG examination was postponed to 5 days after the initiation of osimertinib, which showed atrial fibrillation with third degree atrioventricular block (QTc: 464 ms, HR: 40 bpm, Figure 1B). Then a pacemaker (VVI, Medtronic, E10A1) was implanted in 10 November 2021, and her ECG was normal (QTc: 488 ms, HR: 60 bpm, Figure 1C). In 1 January 2022, 60 days after administration of osimertinib, she had some tea made of leaves and fruits (≈10 g) from TCM named “Litsea Cubeba” due to heatstroke. In 2 January 2022, she suddenly fainted when cooking at home at around 7:00 and woke up approximately 30 min later, feeling a little dizzy and chest tightness. She fainted again when walking around 14:00 and woke up after about 1 h, then she was sent to our hospital for emergency treatment. UCG showed a LVEDD of 57 mm and an LVEF of 57%. The ECG indicated TdP (QTc: 532 ms, HR: 60 bpm, Figure 1D). A loading dose of amiodarone of 0.15 g was intravenously injected immediately, followed by continuous infusion at 1 mg/min. In the meantime, intravenous administration of potassium and magnesium (500 ml 5% GS supplement of 25% magnesium sulfate 10 ml and 10% muriate 15 ml) and omeprazole (40 mg in 0.9% NS 100 ml) were carried out. She was transferred to cardiovascular medicine for further treatment at 20:51.

The vital signs were temperature (T) 36.3°C, blood pressure (BP) 89/69 mmHg, heart rate (HR) 59 bpm, and respiratory rate (RR) 20 bpm during the initial examination. Chest CT on admission showed a little fiber focus in the middle lobe of the right lung and small calcifications in the lower lobe of the right lung. Electrolytes: potassium 3.5 mmol/L, calcium 2.21 mmol/L; blood routine: white blood cells 10.7 × 10^9^/L, neutrophils 9.5 × 10^9^/L; BNP: 248 pg/mL.

Half an hour after admission (21:20), her condition suddenly deteriorated. Tdp (QTc: 515 ms, HR: 110 bpm, Figure 1E) with loss of consciousness occurring repeatedly. Cardiopulmonary resuscitation of repeated chest compressions was carried out. Supplement of potassium and magnesium (15 ml of 10% potassium chloride and 20 ml of 25% magnesium sulfate were added to 500 ml of 5% glucose) were intravenously injected immediately and had a dosage adjustment according to serum electrolytes (Table 1). Meanwhile, 50 mg of lidocaine was intravenously injected at the first dose, resulting in no reduction of premature beats. Then an additional 150 mg was injected, followed by continuous infusion at 1 mg/min. Furthermore, pacemaker frequency was adjusted to 100 bpm and gradually reduced to 70 bpm based on her ECG monitoring (Table 1). Finally, TdP terminated at 22:20 and was not recurrent afterward. As she had >10 year history of stable diabetes, subcutaneous injection of insulin Degludec and insulin Aspart injection (Ryzodeg) was continued. She also had >1 year history of stable hypertension treated with valsartan (80 mg qd po), but valsartan (80 mg qd po) was discontinued due to the low blood pressure during her hospitalization. Her ECG did not return to normal (QTc:528 ms, HR:69 bpm, Figure 1F) at discharge, partly because of amiodarone use. She was discharged on the ninth hospital day.

**TABLE 1:** Serum electrolyte and pacing frequency adjustment in hospital.

Date	Electrolytes			Pacing frequency adjustment (beats/min)
	Potassium (mmol/L)	Magnesium (mmol/L)	Calcium (mmol/L)	
January 2, 2022	3.5	NA	2.21	60
January 3, 2022	5.0	1.64	2.01	100
January 4, 2022	NA	NA	NA	90
January 5, 2022	NA	NA	NA	80
January 6, 2022	4.66	0.83	2.14	80
January 7, 2022	NA	NA	NA	70
January 11, 2022	4.99	0.67	2.40	70

*NA, not available.*

## Discussion

Over the past 10 years, overall survival among cancer patients has been extended due to the development of efficacious treatment regimens, while many of these treatments can cause QTc prolongation (6). QTc prolongation is an unusual but important cardiac adverse reaction of anti-tumor drugs, especially chemotherapeutic agents, EGFR-TKI, and immune checkpoint inhibitors, which can lead to TdP, a potentially life-threatening form of malignant arrhythmia with clinical manifestations characterized by syncope, tetany, or sudden death. In a scientific statement from the American Heart Association and the American College of Cardiology Foundation published in Drew et al. (7), the normal QTc interval is recommended to be 470 ms for males and 480 ms for females. QTc interval >500 ms is considered highly abnormal in both males and females, and may be associated with a substantially elevated risk of TdP (8). Usually, the risk of TdP increase 5∼7% for every 10 ms extention in the QTc interval (9). But the link between changes in QT and TdP is highly variable. For some individuals, TdP may occur with modest QT prolongation, whereas others may experience no effects even with markedly prolonged QT (10). That is because Tdp is also associated with increased dispersion of ventricular repolarization. For example, amiodarone can markedly increase QT, but typically has a homogenous effect on the ventricular myocardium and rarely causes TdP (7, 11).

Osimertinib, an oral, third-generation EGFR-TKI that can specifically bind mutated T790M, has been approved for the treatment of NSCLC patients with T790M mutation. According to a United States retrospective clinical study from 2016 to 2018, osimertinib significantly increased the risk of cardiotoxicity compared with other EGFR-TKIs, and QT prolongation was the second common AE next to cardiac failure (12). In that study, the median onset of QT interval prolonged following osimertinib initiation was 23 days (IQR:14∼55). A Japanese retrospective study of 3,578 patients treated with osimertinib from 2016 to 2018 also reported that QT prolongation was observed in 45 patients (1.3%; Grade ≥3, 0.1% [5/3578]), while other cardiac disorders (including cardiac disorders, cardiac failure, and cardiomyopathy) were observed in 101 patients (2.3%) (13). In that study, the median onset of QT interval prolonged was 56 days (range:4∼548) after the first dose. Most prolonged QTc induced by osimertinib is mild to moderate (grade 1–2) adverse events, as 0.9% patients have prolonged QTc over 500 ms and 3.6% patients has prolonged QTc above 60 ms from baseline in clinical trials (4, 5). As per the osimertinib package insert, if the QT interval is >500 ms the osimertinib should be withheld until the QT interval is <480 ms or recovers to baseline. If the baseline QT is >480 ms, then osimertinib should be resumed at one-half the dose (14). The mechanism of osimertinib-induced QT interval prolongation may associate with potassium channels which plays an important role in repolarization. EGFR are linked to many potassium channels, i.e., Kv1.3 (15), Kv10.1 (16), Kv4.3 (17), and Kir2.3 (18), which are modulated by EGFR kinase *via* phosphorylation of tyrosine in their specific residues. Osimertinib has been proved as a weak inhibitor of the cardiac potassium ion channel, Kv11.1, inhibiting hERG encoded potassium channel function with an *in vitro* IC50 of 0.69 mM (19). Spontaneous release of Ca^2 +^ *via* cardiac ryanodine receptors (RyR2), through a process termed store overload-induced Ca^2 +^ release (SOICR), is a common mechanism underlying arrhythmia. It has been reported that some class I kinase inhibitors (silmitasertib, sunitinib) can increase the activity of RyR2, ultimately increasing the propensity for SOICR (20), which suggests that RyR2 may contribute to arrhythmia induced by Osimertinib. Besides, animal model suggests that down-regulation of PI3K signaling directly or indirectly *via* tyrosine kinase inhibition prolongs the QT interval by affecting multiple ion channels, i.e., decrease the delayed rectifier K^+^ currents (I_*Kr*_ and I_*Ks*_), the L-type calcium ion (Ca^2 +^) current (I_*Ca*,__*L*_) and the peak sodium ion (Na^+^) current (I_*Na*_) and increase the persistent Na^+^ current I_*NaP*_ (21).

No Tdp associated with osimertinib has occurred in clinical trials (4, 5), and only three cases of Tdp caused by osimertinib have been reported (Table 2). In addition, Kondo (25) reported a case of an 85-year-old female patient treated with osimertinib for advanced lung cancer expressing EGFR mutations (T790M), which lengthened QT interval causing abortive sudden cardiac death (SCD). Cardiac arrest in that case was supposed to be due to possible development of TdP based on several predisposing factors lengthening QT interval. However, the case was not presented in Table 2, because the ECG was not retrieved from AED, resulting in no ostensive proof of Tdp. Here we present a 68-year-old woman with advanced NSCLC who underwent QT prolongation leading to Tdp when combined with a TCM named “Litsea Cubeba.” In this case, Tdp happened along with QTc interval prolonged from 464 to 515 ms in 61 days after osimertinib administration.

**TABLE 2:** Review of case reports of Tdp due to osimertinib.

Authors	Age, sex	EGFR mt	Heart diseases	Prior treatment	Osimertinib response	Time to Tdp	QTc				Suspicious concomitant drugs	Tdp outcome
							Baseline	Tdp occurr	Tdp Terminated	Tdp Discharge		
Bain (22)	85, M	L861Q + T790M	ND	Gefitinib	ND	6 months	484 ms	647 ms	631*/496 ms^#^	ND	Moxifloxacin	Improved
Ikebe (23)	84, F	Dell 9	None	SRT	PR	2 months	467 ms	524 ms	ND	464 ms	ND	Improved
Matsuura (24)	60, F	ND	QT prolongation without syncope	ND	ND	2 months	486 ms	532 ms	ND	475 ms^$^	General anesthesia	Improved
This case	68, F	ND	HTN, DB, AF, VVI-IP	Lcotinib	PR	2 months	481 ms	515 ms	510 ms	528 ms	Litsea Cubeba	Improved

AF, atrial fibrillation; DB, diabetes; EGFR mt, epidermal growth factor receptor mutation; F, female; HTN, hypertension; M, male; ND, not documented; PR, partial response; SRT, stereotactic thoracic radiotherapy; TdP, torsade de pointes; VVI-IP, a pacemaker (VVI, Medtronic, E10A1) implanted; *7 h after Tdp occurr, ^#^91 h after Tdp occurr, ^$^12 day after discharge.

Litsea Cubeba is a traditional herb and its plant parts, such as bark, leaf, root, and fruits, have been utilized in TCM for various diseases, including carminative (relieves flatulence), diuretic (aids urine passage), expectorant (aids secretion of sputum), stomachache, antiasthmatic, dyspepsia, gastroenteritis, diabetes, edema, cold, arthritis, pain, traumatic injury, and so on (26). In this case, the patient diagnosed herself with heatstroke based on a series of symptoms such as dizziness, nausea, and vomiting. Then she had some tea made of leaves and fruits (≈10 g) from Litsea Cubeba. Litsea cubeba encompasses a varied number of active compounds, such as alkaloids, monoterpenes, sesquiterpenes, diterpenes, flavonoids, amides, lignans, steroids, and fatty acids. According to TCM systems pharmacology database and analysis platform (TCMSP),^1^ there are at least 11 compounds that can target potassium voltage-gated channel subfamily H member 2 (KCNH2), one of the important potassium channels that regulate vascular tonus and QT interval in ECG. Thus, Litsea Cubeba may affect QT interval and partly contributed to the Tdp.

Besides, risk factors for the development of TdP in hospitalized patients include: QTc >500 ms, use of QT-prolonging drugs, heart disease (congestive heart failure and myocardial infarction), advanced age, female sex, electrolyte disturbance (hypokalemia, hypomagnesemia, hypocalcemia, etc.), treatment with diuretics, impaired hepatic drug metabolism (hepatic dysfunction or drug-drug interactions), bradycardia, genetic polymorphisms, and so on (7). In this case, the patient had at least third risk factors, i.e., advanced age, female sex, concurrent use of more than 1 QT-prolonging drug, history of atrial fibrillation with third degree atrioventricular blocker, might partly contribute to the TdP.

## Conclusion

We have described a 68-year-old female patient with EGFR mutant advanced lung cancer treated with standard regimen of osimertinib, underwent QT prolongation leading to Tdp when combined with a TCM named “Litsea Cubeba.” Magnesium supplementation, potassium supplementation, and the antiarrhythmic drug lidocaine was given, so as the pacemaker frequency was adjusted to 70∼100 bpm. Then, Tdp terminated, but QT prolongation sustained at discharge.

Although osimertinib-induced QT prolongation is mild to moderate and not frequent (3∼10%), TdP may be induced by standard dose of osimertinib, especially when multiple risk factors to lengthen QT interval are incidentally overlapped. Concomitant drugs that can increase QT prolongation should be avoided while taking osimertinib. Besides, we recommend that clinicians consider an ECG at baseline when initiating osimertinib and at periodic intervals to monitor for QT prolongation. Dose reduction or treatment interruption should be performed for serious adverse events of QTc prolongation or even serious arrhythmia.

## Data Availability Statement

The original contributions presented in the study are included in the article/supplementary material, further inquiries can be directed to the corresponding author.

## Ethics Statement

The studies involving human participants were reviewed and approved by the Ethics Committee of Lishui Central Hospital. The patients/participants provided their written informed consent to participate in this study. Written informed consent was obtained from the individual(s) for the publication of any potentially identifiable images or data included in this article.

## Author Contributions

X-YZ contributed to manuscript writing. C-BW, C-XW, and Y-JZ contributed to data collection. LL contributed to the clinical treatment. Y-YZ contributed to the initial concept of the manuscript preparation. W-QT contributed to the review of the manuscript. S-ML contributed to the editing of the manuscript. All authors collaborated and carried out case report manuscript, read, and approved the final manuscript.

## Conflict of Interest

The authors declare that the research was conducted in the absence of any commercial or financial relationships that could be construed as a potential conflict of interest.

## Publisher’s Note

All claims expressed in this article are solely those of the authors and do not necessarily represent those of their affiliated organizations, or those of the publisher, the editors and the reviewers. Any product that may be evaluated in this article, or claim that may be made by its manufacturer, is not guaranteed or endorsed by the publisher.

## Footnotes

1
https://tcmsp-e.com/tcmsp.php
